# Synaptic plasticity through a naturalistic lens

**DOI:** 10.3389/fnsyn.2023.1250753

**Published:** 2023-12-07

**Authors:** Charlotte Piette, Nicolas Gervasi, Laurent Venance

**Affiliations:** Center for Interdisciplinary Research in Biology (CIRB), College de France, CNRS, INSERM, Université PSL, Paris, France

**Keywords:** synaptic plasticity, *in vivo*-like patterns, neuromodulation, learning, memory, spike-timing dependent plasticity, neuroenergetic, body internal states

## Abstract

From the myriad of studies on neuronal plasticity, investigating its underlying molecular mechanisms up to its behavioral relevance, a very complex landscape has emerged. Recent efforts have been achieved toward more naturalistic investigations as an attempt to better capture the synaptic plasticity underpinning of learning and memory, which has been fostered by the development of *in vivo* electrophysiological and imaging tools. In this review, we examine these naturalistic investigations, by devoting a first part to synaptic plasticity rules issued from naturalistic *in vivo*-like activity patterns. We next give an overview of the novel tools, which enable an increased spatio-temporal specificity for detecting and manipulating plasticity expressed at individual spines up to neuronal circuit level during behavior. Finally, we put particular emphasis on works considering brain-body communication loops and macroscale contributors to synaptic plasticity, such as body internal states and brain energy metabolism.

## Background

There is an increasing body of evidence in favor of the neuronal plasticity (synaptic, intrinsic and/or structural) and memory hypothesis (Martin and Morris, 2002; Josselyn and Tonegawa, 2020). Synaptic plasticity rules were first investigated *in vitro*, in which neuronal activity patterns can be exactly controlled. Although some rules have been validated *in vivo*, further clarification is needed on how *in vivo* neuronal activity causes synaptic plasticity. Furthermore, the diversity of plasticity rules and profiles expressed within an individual neuron or a given circuit, the plasticitome (McFarlan et al., 2023), calls for a clearer understanding of their specific functions and also of their interplay during learning. In the light of recent studies, this review aims at highlighting how naturalistic investigations of synaptic plasticity can provide a critical insight into the plasticity and memory research field.

## From neuronal activity patterns to plasticity rules

Multiple plasticity induction protocols, more or less inspired by *in vivo* activity patterns, have been used both *in vitro* and *in vivo* to unveil the spatio-temporal constraints of synaptic plasticity expression in neuronal networks and dissect their molecular determinants. High-frequency stimulation (HFS) is still widely used because it induces reliable and (generally) potent plasticity ((long-term potentiation, LTP, or long-term depression, LTD) LTP or LTD depending on brain areas, neuronal subtypes). Although HFS can somehow mimic some sensory epochs, HFS appears in most conditions rather as an artificial cell conditioning paradigm because of its high and regular stimulation frequency (100 Hz), and duration (typically 1 s repeated several times). Yet, it is crucial to study the effects of stimulation protocols using natural activity patterns, obtained from *in vivo* electrophysiological recordings (Paulsen and Sejnowski, 2000). Indeed, it will inform on which activity patterns are sufficient and effective at inducing plasticity during learning *in vivo*, hence uncovering naturalistic plasticity rules. In addition, it enables to identify molecular determinants (partially different from those recruited by HFS-induced plasticity), that could later be used for manipulating plasticity expression *in vivo*.

First attempts of realistic stimulations came with theta-burst stimulations, determined from *in vivo* recordings of place cells showing theta rhythm linked to memory storage. Later on, *in vivo* recordings of cortical neurons displaying low frequency firing (<5 Hz) and the discovery of backpropagating action potential (bAP), a signal that could bind presynaptic and postsynaptic activity for plasticity induction, led to spike-timing dependent plasticity (STDP) paradigms (Feldman, 2012; Debanne and Inglebert, 2023). Temporally ordered coincident neuronal activity was postulated by Donald Hebb as the critical driver of long-lasting modifications between neurons (Sejnowski, 1999). Its experimental validation came with the discovery that repeated presynaptic activity preceding post-synaptic activity within a few tens of milliseconds could induce LTP, while the converse temporal order led to LTD; aka Hebbian STDP. Since then, multiple polarity and forms of STDP have been described (Feldman, 2012). Classically, STDP is induced with 100–150 presynaptic and postsynaptic pairings at low frequency (1–2 Hz). However, various forms of STDP aiming at mimicking more *in vivo*-like activity (Debanne and Inglebert, 2023) were also evoked using smaller number of pairings (5–30) (Froemke et al., 2006; Cui et al., 2015, 2016; Cepeda-Prado et al., 2022) as expected in single-trial or one-shot learning (Piette et al., 2020), more complex spiking sequences, such as spike triplets or quadruplets (Froemke and Dan, 2002; Mendes et al., 2020), or *in vivo* spiking patterns replayed between two neighboring neurons *in vitro* (Isaac et al., 2009). STDP was also translated *in vivo* by associating natural sensory stimulation that activates afferents combined with evoked or spontaneous spiking of a single cortical neuron (Yao and Dan, 2001; Meliza and Dan, 2006; Jacob et al., 2007).

**Figure 1:**
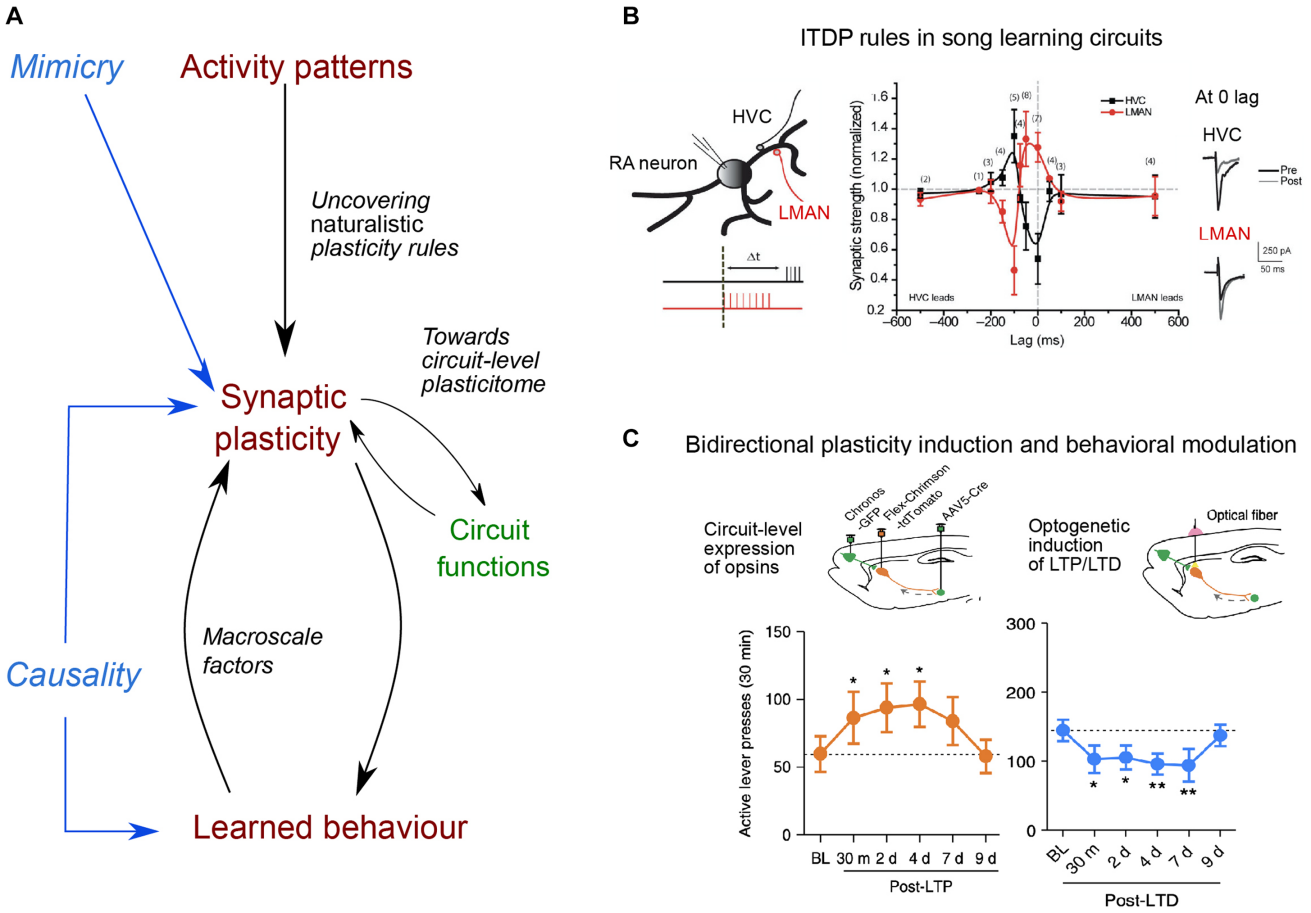
Main directions of investigation for uncovering naturalistic plasticity rules, their circuit functions and causal link to learned behavior. **(A)** Uncovering naturalistic synaptic plasticity rules needs to rely on *in vivo* activity patterns, by considering the rate and timing of naturally incoming inputs, which can then be replayed *in vitro*. Mimicry experiments aim to mimic naturalistic stimulation, compatible with synaptic plasticity expression. Next, understanding the function of synaptic plasticity expression in a given circuit remains a major challenge. Different forms of plasticity, occurring at different synapses, expressed at different timescales and relying on different molecular cascades (constituting the plasticitome) may be evoked within a single event and hence requires a circuit-level investigation, that further considers the presence of third factors (e.g., neuromodulators, neuropeptides or glial cells), and also of macroscale factors (such as body–brain internal states or neuroenergetic load). Finally, determining causal links between synaptic plasticity and learned behavior has remained limited to stereotypical behavior. **(B)** Example of naturalistic stimulation, based on singing-related activity, leading to opposing heterosynaptic plasticity in the songbird cortex (robust nucleus of the arcopallium, RA), in which two inputs critical for song learning converge: afferences from premotor HVC (dark) and one from LMANN (red): when one input is potentiated, the other is depressed, and direction of plasticity (*y*-axis) shows a strong dependence on the relative timing of stimulation (lag, *x*-axis). Example traces of excitatory post-synaptic potentials (EPSC) pre and post-stimulation are displayed for 0 ms time lag. Adapted from Mehaffey and Doupe (2015). **(C)** Example of an evoked-bidirectional plasticity and induced-behavioral changes. LTP or LTD were induced at cortico-striatal synapses *in vivo*, using optogenetic tools and virally-mediated expression of excitatory opsins in medial prefrontal cortex and dorsomedial striatum (top schemas). Induction of LTP and LTD led rats trained to self-administer ethanol to actively press more the lever over several days post-plasticity induction protocol, leading to increased ethanol intake (orange trace), or long-lasting reduction of their number of lever presses (blue trace) their number of lever presses (blue trace), respectively. Adapted from Ma et al. (2018).

STDP rules usually rely on the repetition of precisely timed presynaptic and postsynaptic spikes *in vitro*. Yet the exact contribution of spike timing relative to firing rate in eliciting synaptic plasticity *in vivo* is still debated (Graupner et al., 2016). Related to this, it was shown that plasticity rules vary across cerebellar regions, with a precise time interval of 120 ms between parallel fiber and climbing fiber inputs allowing for plasticity expression in the flocculus (known to receive error signals at this delay during oculomotor learning), while a broader range of intervals are permissive for plasticity expression in the vermis, implicated in a wider variety of learning paradigms (Suvrathan et al., 2016). In addition, cortico-striatal STDPs show different sensitivity to spike timing jitter *in vitro*: endocannabinoid-dependent plasticities (endocannabinoid-LTD and endocannabinoid-LTP) are more robust to spike timing variability compared to NMDA-LTP (Cui et al., 2018). Such differential sensitivities between endocannabinoid-LTP and NMDA-LTP (induced by few pairings, 5–15, versus 100 pairings, respectively) could underlie different functions, at different stages of learning as sequential activity patterns become more and more stereotyped (Thorn et al., 2010; Figure 1).

Furthermore, STDP faces two major interrogations questioning its physiological relevance: (i) Can plasticity only be induced by a global feedback signal, such as a bAP, resulting from the activation of a critical number of synapses? This would contradict the fact that a limited number of synapses could be subjected to plasticity. Also, this implies that only digital (bAP) but not analog signals induce plasticity; (ii) How can the compressed timescale (typically, pairings intervals are <50 milliseconds and repeated at 1 Hz) in which STDP occurs be compatible with behavioral timescales (subseconds to minutes)? Indeed, if STDP is sensitive to correlations of tens or hundreds of milliseconds (Feldman, 2012), temporal associations between different stimuli during learning are typically in the subsecond/minute range (Drew and Abbott, 2006).

The first point has been addressed in different ways. Paired subthreshold events can induce long-term plasticity, such that bAP would not be necessary for plasticity expression (Fino et al., 2009; Brandalise and Gerber, 2014) and strong post-synaptic depolarization was shown to induce sufficient intracellular Ca^2+^ rise, due to dendritic non-linearities, to evoke long-term plasticity (Holthoff et al., 2004; Hardie and Spruston, 2009). Therefore, digital (bAPs) as well as analog (EPSPs) signals can induce *in vitro* plasticity. Because bAPs are attenuated differently among neuronal subtypes, and along the dendritic arbor, in part depending on the level of excitatory and inhibitory synaptic inputs, their contribution is likely to vary *in vivo* (Waters and Helmchen, 2004). Hence, delimiting the exact role and spatial extension of digital and analog signals for triggering synaptic plasticity *in vivo* requires further investigation. This consideration also led to the study of more naturalistic forms of STDP, dependent upon the temporal correlation between two distinct presynaptic afferences, the input-timing-dependent plasticity (ITDP), using timing rules that mapped synaptic delays caused by neural network architecture (Dudman et al., 2007; Cho et al., 2012; Basu et al., 2013; Mehaffey and Doupe, 2015; Leroy et al., 2017; Figure 1). ITDP can be viewed as a physiological upgrade of STDP since it does not rely on an artificial post-synaptic injection of current necessary to create the bAP (see the critical view of STDP in Lisman and Spruston, 2010), but on paired activation of presynaptic inputs triggering sub- or suprathreshold activity in the postsynaptic element.

Regarding the second point, even though the compressed (milliseconds) timescale of STDP is compatible with replay episodes in sleep for learning specific temporal sequences (Debanne and Inglebert, 2023; George et al., 2023), the search for factors contributing to enlarge its temporal window put a strong emphasis on nonlinear slow-timescale mechanisms (e.g. CaMKII and calcineurin) (O’Donnell, 2023) and on the three-factor learning rule (Frémaux and Gerstner, 2016; Edelmann et al., 2017; Foncelle et al., 2018; Brzosko et al., 2019). Indeed, STDP has been originally described as a two-factor rule relying on paired activity in the presynaptic and postsynaptic elements (two-factor) to fulfill Hebb’s postulate, and was later augmented to a third-factor rule to include neuromodulators (e.g. dopamine, noradrenaline or acetylcholine), neurotransmitters (GABA or endocannabinoids), neuropeptides (BDNF) or glia (astrocytes), which efficiently modulate plasticity and can enlarge the temporal window of STDP expression. Indeed, repeated co-activation of synaptic activities, together with dopamine release, leave eligibility traces for about 1 s at cortico-striatal synapses (Yagishita et al., 2014), 5 s in the neocortex (He et al., 2015) or even up to 10 min in the hippocampus (Brzosko et al., 2015; Fuchsberger et al., 2022).

Lastly, a naturalistic plasticity rule was recently uncovered at CA3-CA1 synapses, both *in vitro* and *in vivo*, which no longer requires repetitions and co-activation of presynaptic and postsynaptic elements (Bittner et al., 2017; Priestley et al., 2022; Fan et al., 2023). Synaptic inputs from CA3 place cells are potentiated by the occurrence of a single Ca^2+^ dendritic plateau, produced at distal dendrites. Importantly, the temporal overlap between the two signals can span the second timescale and their temporal order does not impact plasticity expression. The dendritic plateau potential appears as an instructive signal, evoked by specific circumstances (reduced dendritic inhibition, permissive neuromodulatory signaling, strong inputs), occurring for instance during exploration of a novel environment (Priestley et al., 2022) or of a context in which specific task-related information is carried by a given position (Zhao et al., 2022). Due to behavioral timescale plasticity (BTSP) asymmetric time course (Bittner et al., 2017; Magee and Grienberger, 2020), predictive information might be encoded.

This overview presented several refinements aiming at approaching naturalistic synaptic plasticity rules. This effort should be continued, especially since our understanding of synaptic plasticity rules remains mostly defined at the scale of an entire synaptic pathway and focuses on a given population of excitatory or projecting neurons. In this direction, considering interactions between neighboring neurons has unveiled a variety of heterosynaptic plasticity mechanisms (Chistiakova et al., 2015; Mendes et al., 2020). These can influence the net plasticity outcome of a given circuit when interactions between inhibitory and excitatory neurons are examined (D’Amour and Froemke, 2015; Hiratani and Fukai, 2017). Heterogeneities in plasticity expression at the neuronal level can arise from a neuron’s prior and ongoing activity (Han et al., 2007) or its dendritic architecture, in particular the distribution of active inhibitory and excitatory synapses (Harvey and Svoboda, 2007; El-Boustani et al., 2018) or compartmentalized changes in dendritic excitability (Losonczy et al., 2008). These additional considerations reinforce the need to detect input-specific signals from both somatic and dendritic compartments, that could serve as proxies for local synaptic plasticity expression.

This overview also pointed out the importance of reinforcing the translation between *in vivo* and *in vitro* recordings, keeping in mind discrepancies relative to ionic composition (Inglebert et al., 2020), metabolic substrates (Dembitskaya et al., 2022), neuromodulator concentrations or spontaneous activity levels, as well as body-brain internal states, which constitute key factors affecting plasticity induction thresholds, as will be discussed below.

## Toward the uncovering and manipulation of a learning-induced plasticitome

In parallel to extracting synaptic plasticity rules evoked by natural *in vivo*-like activity patterns, a vast number of studies has uncovered learning-induced synaptic changes, hence directly examining synaptic plasticity in naturalistic settings. Detection of synaptic changes historically relied on *in vivo* electrophysiological recordings of synaptic efficacy using electrical stimulation yet lacking cell-type specificity and often restricted to a single circuit, or from *ex vivo* saturation/occlusion experiments or measures of AMPA/NMDAR ratio, which cannot provide a full account of synaptic temporal dynamics. The development of optical stimulation combined with spatial- and cell-specific expression of opsins partially lifted the first limitation: as an example, cortico-striatal plasticity monitored *in vivo* during an auditory discrimination task, based on the selective optogenetic stimulation of cortical neurons along the tonotopic axis, revealed spatially selective plasticity induction depending on reward contingencies (Xiong et al., 2015). Combined with c-Fos labeling of both presynaptic and postsynaptic cells active during fear conditioning, *ex vivo* recordings showed an occlusion of LTP between engram cells, along with changes in presynaptic release probability (Choi et al., 2018). Notably, the all-optical approach combined with imaging of subthreshold membrane potential dynamics and opto-stimulation of afferences allows plasticity detection at the circuit-level (Fan et al., 2023). It could further be extended, by probing multiple regions simultaneously using novel imaging tools, such as light beads microscopy, enabling large volumetric recording of neuronal activity (Demas et al., 2021). In addition, a key advantage of optical approaches is their combinatorial power, by juxtaposing the dynamics of optical sensors and labeling of active cells during behavioral tasks. Typically, to better capture the full temporal dynamics of synaptic plasticity expression, calcium and voltage sensors, which can track initial signatures of synaptic changes, could be combined with sensors related to downstream cascades such as CamKII (Lee et al., 2009) or PKA (Gervasi et al., 2010), which inform on long-term plasticity expression and maintenance *in vivo*. Furthermore, the development of presynaptic vesicular release sensors, which are currently effective *in vitro* (Ferro et al., 2017) should also help investigating the often-neglected presynaptic plasticity loci. Although these tools offer unprecedented access to detailed naturalistic plasticitomes, they also have their own limitations and caveats. Indeed, the expression of opsins combined with viral vectors is not without cell specificity confounds and toxicity-related issues (Miyashita et al., 2013). In addition, opsins or fluorescent sensors can alter natural synaptic dynamics, depending for instance on their expression levels (Jackman et al., 2014).

Chronic tracking of structural dynamics in spine numbers and shapes using *in vivo* 2-photon imaging (Pfeiffer et al., 2018), i.e. structural plasticity, can be used as proxy for synaptic strength (Holtmaat and Svoboda, 2009). Dual-eGRASP, a split fluorescent protein that emits fluorescence only when presynaptic and postsynaptic eGRASP components are physically attached in the synaptic cleft (Choi et al., 2018, 2021), combined with the *Fos* promoter-driven tetracycline transactivator system (Mayford and Reijmers, 2015), allows to track longitudinally *in vivo* synapses between pre and post-synaptic neurons active or not during learning (Lee et al., 2023). Other avenues, down to *in vivo* tracking of receptor dynamics, have also been opened (Matsuo et al., 2008; Zhang et al., 2015) and can be envisaged simultaneously across thousands of synapses (Graves et al., 2021).

Beyond the detailed characterization of plasticity expression *in vivo* during learning, the demonstration of a causality between plasticity expression and learned behavior now represents a current grail in neurophysiology, such that bidirectional behavioral modifications can be caused by bidirectional manipulation of synaptic efficacy. Currently, only a couple of studies has achieved such bidirectional control: a conditioned fear response was, respectively, erased and restored upon depotentiation and re-potentiation of the auditory inputs to the lateral amygdala (Nabavi et al., 2014). Likewise, cortico-striatal opto-induced-LTP and -LTD promoted and decreased, respectively, alcohol-seeking behavior (Ma et al., 2018; Figure 1). Optogenetically-induced depotentiation of LTP, initially induced by auditory fear conditioning, suppressed fear responses to the conditioned stimulus (Kim et al., 2007). Furthermore, with two auditory stimuli underlying two different memories, opto-potentiation and -depotentiation of synapses shared within each specific cell assembly selectively restored or impaired the retrieval of one memory while sparing the other (Abdou et al., 2018). These causal manipulations should now aim at triggering reversible synaptic changes using naturalistic plasticity induction protocols, instead of classical low or high-frequency stimulation. In addition, to further nail down causality at the synapse-level, a specific ChR2 expression on recently activated synapses could allow more physiological excitation, by mimicking *in vivo* occurring calcium transients, compared to full somatic activation (Gobbo et al., 2017). In the future, one could even imagine modulating bidirectionally and reversibly the excitability of individual spines or dendritic branches during learning, for instance through targeted expression of both hyperpolarizing and excitatory opsins. Yet, an ongoing issue of manipulating synaptic efficacy relates to its specificity, and the absence of interference with other synaptic mechanisms or basal neurotransmission, as well as the possibility to cause other pathological changes or evoke compensatory mechanisms. Therefore, the development of spatio-temporally precise manipulations, with sensor expression impacting the least physiological dynamics, remains of critical importance.

Finally, instead of directly manipulating synaptic weights to cause behavioral changes, mimicry experiments currently (and somehow paradoxically) offer more naturalistic settings for testing the memory and synaptic plasticity hypothesis. Indeed, mimicry consists of artificially stimulating neuronal circuits *in vivo* (without undergoing any kind of experience) and triggering behavioral changes. The stimulation mimics putative activity patterns during a real learning experience and can therefore bridge naturalistic synaptic plasticity rules described above and their behavioral relevance. At this day, only associations between conditioned and unconditioned stimuli were mimicked. Building up on previous works in which a partial sensory experience combined with opto-stimulation created artificial memories (Josselyn and Tonegawa, 2020), an artificial memory was generated by combining patterned stimulation of olfactory glomeruli with the stimulation of distinct inputs to the ventral tegmental area that mediated either aversion or reward (Vetere et al., 2019). A next challenge will be to move from neuronal assemblies down to the synaptic level in these same simple behavioral paradigms (using tools described above; Gobbo et al., 2017), and to generalize to more complex learning, using naturalistic sequences of neuronal activation.

## The (almost) overlooked of synaptic plasticity research: the body–brain communication loops and neuroenergetics

In synaptic plasticity, besides the two “Hebbian” factors (presynaptic and postsynaptic activities), a third “neoHebbian” factor allows the stabilization and shaping of plasticity maps. This third factor gathered well-defined elements such as neurotransmitters/neuromodulators, neuropeptides, fatty acids or glial cells (reviewed in: Frémaux and Gerstner, 2016; Foncelle et al., 2018; Brzosko et al., 2019). Here, we chose to focus on macroscale factors, defined by integrated body–brain communication loops, also in relation to external states (Kanwal et al., 2021; Flavell et al., 2022). Indeed, the brain receives massive sensorimotor feedback from the body, such as heartbeat (Hsueh et al., 2023), blood pressure, respiratory rate (Folschweiller and Sauer, 2023), gastric fullness, internal temperature or visceral pain. During active behavioral states, the integration of these feedbacks engages widespread circuits. Macroscale factors also include sleep–wake, circadian or seasonal-related rhythms, as well as metabolic (thirst and feeding) states. These factors are mediated by brain–body endocrine communication, metabolic substrates and associated signaling molecules, and recruit neuromodulators, glial or immune cells. As an illustration, the general body state changes during exercise can favor plasticity expression. Weak theta-pattern stimulation of the hippocampus, which does not produce LTP in control rats, induces LTP in rats housed with a running wheel (Farmer et al., 2004). Voluntary exercise, by increasing theta oscillation and lowering LTP induction threshold, may prime the network to promote synaptic plasticity *in vitro* and *in vivo* (van Praag et al., 1999).

Secretory molecules and vesicles released by organs such as skeletal muscle, adipose tissue, liver and gut are part of the body–brain feedback (Pedersen and Febbraio, 2012). These molecules, such as FNDC5/irisin, adiponectin, or IL-6, cross the blood–brain barrier, (i) induce changes in neurotrophins such as BDNF or EGF-1, associated with improvements in hippocampus plasticity, spatial memory, and object recognition (Vaynman et al., 2004; Gomes da Silva et al., 2010), (ii) modulate the cerebrovasculature, allowing improvements in energy metabolism, delivery of oxygen, nutrients, neurotrophins and other factors promoting learning and memory, and (iii) act on plasticity through the increased number, cell body size and arborization length of astrocytes, (Saur et al., 2014), impairment of microglia (Vukovic et al., 2012) and increased neurogenesis (van Praag et al., 1999).

**Figure 2:**
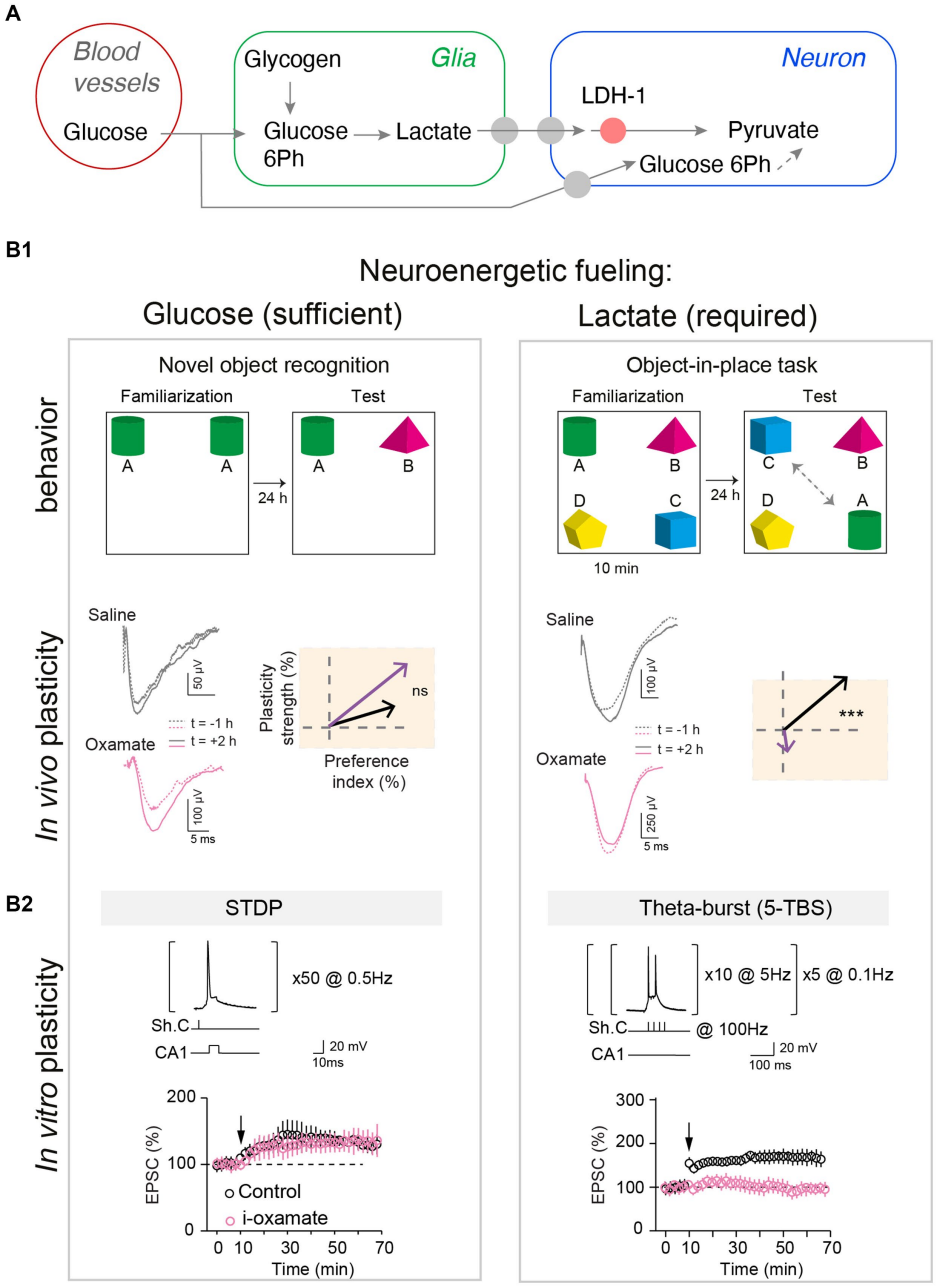
Glucose and lactate metabolisms are differently engaged in neuronal fueling for plasticity expression and memory. **(A)** Main steps of the glucose and the glia-derived lactate transports: astrocytic glycogen catalysis into glucose-6-phosphate and then lactate, lactate entry in neurons via monocarboxylate transporters, and lactate conversion into pyruvate by the neuronal lactate dehydrogenase (LDH-1). **(B1)** Lactate metabolism is necessary for learning cognitive tasks requiring high attentional load as exemplified in the object-in-place task (with four objects) and for expressing the corresponding *in vivo* hippocampal LTP, but glucose is sufficient for a less demanding task such as a simple novel object recognition (with two objects). Rats were injected bilaterally, via cannulas implanted above hippocampal CA1 layer, with either saline or oxamate (50 mM), an inhibitor of the neuronal LDH preventing the conversion of lactate into pyruvate, before familiarization step. Rats with saline performed equally well in both tasks whereas rats receiving oxamate did not detect novelty in the object-in-place task (illustrated by a low preference index value) and did not express LTP (averaged vectors: *y*-axis indicates LTP versus LTD expression and *x*-axis the learning performance evaluated with the preference index). *In vivo* synaptic plasticity during behavioral task with evoked-field-EPSP recorded before familiarization (baseline) and 2 hours after familiarization to determine synaptic changes, in relation with behavior. **(B2)** Lactate metabolism is mandatory to fuel the demanding neural computations implicated in NMDA receptor-mediated LTP forms in hippocampus triggered by theta-burst stimulations, while glucose metabolism is sufficient for lighter forms of LTP, based on less and lower-frequency stimulations. The structure of the plasticity induction protocols and the averaged time-course of the synaptic weight after theta-burst stimulation and STDP protocols are illustrated. Oxamate was applied intracellularly (via the patch-clamp pipette) in the sole recorded neuron, and LDH inhibition shows distinct effects on theta-burst stimulation and STDP expression since it prevented theta-burst stimulation-induced LTP but not STDP-induced LTP. In conclusion, scaling of the computational and cognitive loads requires the metabolism of glia-derived lactate to match the neuroenergetic needs of sustained neuronal activity patterns and high cognitive load, and for less demanding plasticity and learning paradigms, glucose suffices as an energy substrate. Adapted from Dembitskaya et al. (2022).

Among the macroscale factors that could control plasticity expression, neuroenergetics has received particular attention. Synaptic activity is the most energy-consuming process in the brain (Attwell and Laughlin, 2001). Synaptic energy supply is provided on-demand (Kasischke et al., 2004; Chuquet et al., 2010; Ruminot et al., 2017) by neuronal glycolysis and/or glial-derived lactate (via the astrocyte-neuron lactate shuttle) (Magistretti and Allaman, 2018; Bonvento and Bolanos, 2021). Synaptic plasticity and learned behavior depend on the metabolic reservoir. This is well illustrated under food restriction, in flies, with a trade-off between long-term memory establishment and survival (Mery and Kawecki, 2005; Plaçais and Preat, 2013). Long-term neuronal reconfigurations, leading to behavioral changes, mediated by feeding state have also been identified in *C. elegans* (Takeishi et al., 2020) and in the Etruscan shrew (Ray et al., 2020). In addition, top-down adaptations have also been identified between synaptic plasticity induction and energy uptake mechanisms following learning, with vascular adaptations (Lacoste et al., 2014), glial recruitment (Genoud et al., 2006), altered expression of insulin-sensitive glucose or lactate transporters (Tadi et al., 2015; Ashrafi et al., 2017) and mitochondrial activity (Todorova and Blokland, 2017). Interestingly, flies increase their energy intake following multiple trials training, leading to a dopamine-mediated upregulation of cellular metabolism driving LTP in the brain region involved in long-term memory (Plaçais et al., 2017). Dissecting precisely how the energy available and the nature of metabolic substrates – especially lactate and/or glucose- can control synaptic plasticity expression has been the subject of several works (Newman et al., 2011; Suzuki et al., 2011; Murphy-Royal et al., 2020); of note, lactate is also a signaling metabolite and as such acts on neuronal excitability and plasticity via NMDA receptors and or hydroxycarboxylic acid receptor type-1 (HCAR1) (Magistretti and Allaman, 2018). In particular, during learning, the neuronal computational load at play may be more or less intense, raising the possibility that plasticity induction could be more or less metabolically demanding and therefore requires different metabolic pathways. Indeed, lactate supply was required for high stimulation load activity patterns (theta-burst-induced LTP) in CA3-CA1 circuit, whereas glucose was sufficient for less demanding neural computation (low-frequency STDP paradigm) (Dembitskaya et al., 2022). Interestingly, this switch in metabolic substrates was also visible *in vivo* when novel object exploration required a higher attentional and cognitive load and for the corresponding *in vivo* LTP expression (Dembitskaya et al., 2022; Figure 2). It remains to examine how glucose and lactate intervene as exclusive or combined fueling in various engrams depending on the body internal states (diet, emotions, effort) and how global energy is regionally redistributed to meet cellular metabolism (Bruckmaier et al., 2020). More generally, investigating how synaptic plasticity induction and maintenance can be controlled by specific diets (ketone-based or high-fat diet) and is altered in various metabolic diseases (obesity, diabetes, but also neurodegenerative diseases) might provide further mechanistic understanding of body–brain interactions. Yet, as these body–brain communication loops and macroscale factors usually fluctuate on slower timescales relative to synaptic activation, evidence for their causal interplay might be particularly challenging to achieve.

## Conclusion

This mini-review presented key avenues, initiated in the synaptic plasticity and memory research field, that put forward a naturalistic viewpoint. This naturalistic lens was first directed at presenting naturalistic synaptic plasticity rules, based on *in vivo* neuronal activity patterns recorded during learning experience, which can then be dissected *in vitro* and/or *in vivo*. Next, it focused on current advances for uncovering naturalistic plasticitomes, i.e. induced by the animal’s own experience, which can provide detailed spatio-temporal characterizations of synaptic plasticity. Finally, besides well-defined third factors (neuromodulators, neuropeptides or glia), this-mini review emphasized that macroscale factors (internal states and the neuronal energy fueling with glucose and lactate metabolisms) can interplay with synaptic plasticity, and hence participate in defining a complex naturalistic context that shapes synaptic plasticity expression during behavior. The next challenges will be to further nail down the relevant synaptic plasticity rules and associated signaling cascades engaged *in vivo*, by investigating causal interactions between neuronal activity patterns, plasticity maps and behavioral consequences. To further enlarge our naturalistic lens on synaptic plasticity, feasibility of mimicry and causality demonstrations should be tested on complex and natural behaviors, such as episodic-like memory or procedural learning. More attention should also be drawn to the existence and contribution of macroscale factors with the major difficulty of their inextricable bounds to natural behaviors and causal manipulations.

## Author contributions

CP wrote the first draft of the “From neuronal activity patterns to plasticity rules” and “Toward the uncovering of a learning-induced plasticitome” sections, and design the Figure 1. LV and NG wrote the “The (almost) overlooked of synaptic plasticity research: the body–brain communication loops and neuroenergetics” section. LV redraft the whole manuscript, and design the Figure 2. All authors wrote “background” and “conclusions” sections, and have edited and corrected the manuscript.
